# Expression of proteins related to autotaxin–lysophosphatidate signaling in thyroid tumors

**DOI:** 10.1186/s12967-019-2028-7

**Published:** 2019-08-28

**Authors:** Eunah Shin, Ja Seung Koo

**Affiliations:** 1 Department of Pathology, CHA Gangnam Medical Center, CHA University School of Medicine, Seoul, South Korea; 2 Department of Pathology, Yonsei University College of Medicine, Severance Hospital, 50 Yonsei-ro, Seodaemun-gu, Seoul, 120-752 South Korea

**Keywords:** Autotaxin, Lysophosphatidate, Thyroid, Tumor

## Abstract

**Background:**

We aimed to investigate the expression of proteins related with autotaxin (ATX)–lysophosphatidate (LPA) signaling and the clinical implications in primary and metastatic thyroid tumors.

**Methods:**

We constructed tissue microarrays with 545 primary thyroid tumors [338 papillary thyroid carcinoma (PTC), 111 follicular carcinoma (FC), 69 medullary carcinoma (MC), 23 poorly differentiated carcinoma (PDC), and four anaplastic carcinoma (AC)]. Immunohistochemical stains for proteins related to ATX–LPA signaling (e.g., ATX, LPA1, LPA2, and LPA3) were performed.

**Results:**

The expression of ATX was highest in MC, while the LPA1 expression was higher in PDC and AC, and the expression of LPA2 and LPA3 was highest in PTC (p < 0.001). Additionally, the expression of ATX, LPA1, and LPA2 was higher in conventional-type PTC than in follicular-variant PTC (p < 0.05). PTC with BRAF V600E mutation showed higher expression of ATX, LPA1, LPA2, and LPA3 than PTC without BRAF V600E mutation (p < 0.001). In univariate analysis, ATX positivity (p = 0.005) and LPA1 positivity (p = 0.014) were correlated with shorter overall survival in PTC.

**Conclusion:**

Proteins related to the ATX–LPA axis showed different levels of expression in primary thyroid tumors according to subtype.

## Background

Autotaxin (ATX) is a glycoprotein transcribed by the ENPP2 gene on chromosome 8 [1]. ATX is the same molecule as lysophospholipase D and converts lysophosphatidylcholine (LPC) to bioactive lipid mediator lysophosphatidate (LPA). LPA, after binding to the appropriate receptor, activates phospholipase C and the MAPK, PI3K, and RhoA pathways and is involved in various cellular processes [2, 3]. LPA receptor is a G-protein-coupled receptor. There are at least six LPA receptors, LPA1 through LPA6. LPA1 through LPA3 belong to the EGD family (i.e., LPA1-EDG2, LPA2-EDG4, LPA3-EDG7), while LPA4 through LPA6 are similar to the P2Y nucleotide receptor [4]. This ATX–LPA signaling is involved in tumor formation, progression, and metastasis [5, 6].

Thyroid cancer is a relatively common malignancy, affecting about 1% of the population. The most common histologic type of thyroid cancer is papillary thyroid carcinoma (PTC), followed by follicular carcinoma (FC), medullary carcinoma (MC), poorly differentiated carcinoma (PDC), and anaplastic carcinoma (AC). These histologic subtypes of thyroid cancer are known to have different cell origins, clinical manifestations, metastatic patterns, and clinical prognoses. Previously, ATX has been reported to be highly expressed in undifferentiated carcinoma [7], and it is also indicated to be expressed in follicular carcinoma [8]. Furthermore, as ATX expression is higher in thyroid cancer than in benign lesion [9] and is correlated with prognostic factors in thyroid cancer [10], ATX–LPA signaling is likely to have an influence on thyroid cancer biology. However, studies on proteins related to ATX–LPA signaling in recurrent and metastatic thyroid cancer and according to thyroid tumor subtype have not been reported. Thus, we aimed to assess the expression of proteins related to ATX–LPA signaling in recurrent and metastatic thyroid cancer and according to thyroid tumor subtype.

## Materials and methods

### Patient selection and histologic evaluation

Patients diagnosed with PTC who underwent surgery between January 2012 and December 2013 at Severance Hospital, Sinchon-dong, South Korea, were included in the study. As for the remaining subtypes, patients who underwent surgery between January 2000 and December 2014 at Severance Hospital were included. Those who had received preoperative chemotherapy were excluded. This study was approved by the Institutional Review Board of Yonsei University Severance Hospital. Hematoxylin-and-eosin-stained slides of all cases were retrospectively reviewed by a thyroid pathologist (Koo, JS). Clinicopathologic data were obtained from the patients’ medical records and included age at diagnosis, disease recurrence, metastasis, current status, and length of follow-up. Tumor size, location (right or left lobe), extent (confined to the thyroid parenchyme or with extrathyroidal spread), and number of metastatic lymph nodes were also noted from a review of the slides and the surgical pathology reports.

Tumor stroma of PTC was classified as follows: desmoplastic type, tumor stroma composed of cellular fibroblast/myofibroblast proliferation; sclerotic type, tumor stroma composed of collagenous component with few cellular components; pauci type, near absence of a stromal reaction; or inflammatory type, tumor stroma composed of inflammatory cells such as lymphocytes.

### Tissue microarray

A representative area showing tumor and tumor stroma was selected on a hematoxylin-and-eosin-stained slide, and a corresponding spot was marked on the surface of the paraffin block. Using a biopsy needle, the selected area was punched out, and a 3-mm tissue core was transferred to a 6 × 5 recipient block. Two tissue cores from the invasive tumors were extracted to minimize extraction bias. Each tissue core was assigned a unique tissue microarray location number that was linked to a database containing other clinicopathologic data.

### Immunohistochemistry

The antibodies used for immunohistochemistry in this study are shown in Table 1. Formalin-fixed, paraffin-embedded tissue sections were used for immunohistochemistry. During the experiments, 3-μm-thick tissue sections were deparaffinized and rehydrated in xylene and graded alcohol. We used the Ventana Discovery XT automated stainer (Ventana Medical Systems, Tucson, AZ, USA). Cell Conditioning 1 buffer (citrate buffer, pH: 6.0; Ventana Medical Systems, Tucson, AZ, USA) was used for antigen retrieval. Appropriate positive and negative controls were used for each antibody.

**Table 1:** Expression of ATX–LPA axis-related proteins according to the histologic subtype of thyroid cancer

Parameters	Total n = 545 (%)	PTC n = 338 (%)	FC n = 111 (%)	MC n = 69 (%)	PDC n = 23 (%)	AC n = 4 (%)	p-value
ATX							*<* *0.001*
Negative	368 (67.5)	220 (65.1)	102 (91.9)	22 (31.9)	21 (91.3)	3 (75.0)	
Positive	177 (32.5)	118 (34.9)	9 (8.1)	47 (68.1)	2 (8.7)	1 (25.0)	
LPA1							*<* *0.001*
Negative	292 (53.6)	169 (50.0)	73 (65.8)	44 (63.8)	5 (21.7)	1 (25.0)	
Positive	253 (46.4)	169 (50.0)	38 (34.2)	25 (36.2)	18 (78.3)	3 (75.0)	
LPA1 (S)							0.051
Negative	530 (97.2)	323 (95.6)	111 (100.0)	69 (100.0)	23 (100.0)	4 (100.0)	
Positive	15 (2.8)	15 (4.4)	0 (0.0)	0 (0.0)	0 (0.0)	0 (0.0)	
LPA2							*<* *0.001*
Negative	365 (67.0)	191 (56.5)	85 (76.6)	69 (100.0)	16 (69.6)	4 (100.0)	
Positive	180 (33.0)	147 (43.5)	26 (23.4)	0 (0.0)	7 (30.4)	0 (0.0)	
LPA3							*<* *0.001*
Negative	189 (34.7)	56 (16.6)	52 (46.8)	67 (97.1)	10 (43.5)	4 (100.0)	
Positive	356 (65.3)	282 (83.4)	59 (53.2)	2 (2.9)	13 (56.5)	0 (0.0)	

Italic values represent significance of p-value (p < 0.05)

*PTC* papillary thyroid carcinoma, *FC* follicular carcinoma, *MC* medullary carcinoma, *PDC* poorly differentiated carcinoma, *AC* anaplastic carcinoma

### Interpretation of immunohistochemical staining

Immunohistochemical markers were assessed by light microscopy. All stained slides were semi-quantitatively evaluated [11]. Tumor and stromal cell staining was assessed as 0: negative or weak immunostaining in < 1% of the tumor/stroma; 1: focal expression in 1% to 10% of the tumor/stroma; 2: positive in 11% to 50% of the tumor/stroma; or 3: positive in 51% to 100% of the tumor/stroma. Entire tumor areas included in the TMA were evaluated in all cases; a grade of 0 was considered negative and grades of 1 to 3 were considered positive.

### Statistical analysis

Data were analyzed using Statistical Package for the Social Sciences for Windows, version 23.0 (SPSS Inc., Chicago, IL, USA). For determination of statistical significance, Student’s *t* test and Fisher’s exact test were used for continuous and categorical variables, respectively. When analyzing data with multiple comparisons, a corrected p-value with application of the Bonferroni multiple comparison procedure was used. Statistical significance was set at p < 0.05. Kaplan–Meier survival curves and log-rank statistics were employed to evaluate the time to tumor recurrence and overall survival. Multivariate regression analysis was performed using the Cox proportional hazards model.

## Results

### Basal characteristics of thyroid cancer

All retrieved cases were subject to this study, consisting of 338 cases of PTC, 111 cases of FC, 69 cases of MC, 23 cases of PDC, and four cases of AC. Basal characteristics of PTC are listed in Additional file 1: Table S3. PTC cases were composed of 302 conventional type and 36 follicular variant, and 236 cases of PTC (69.8%) had BRAF V600E mutation. FC consisted of 61 cases of minimally invasive type, 37 cases of encapsulated angioinvasive type, and 13 cases of widely invasive type (Additional file 1: Table S3). Basal characteristics of MC, PDC, and AC are listed in Additional file 1: Table S3.

### Expression of proteins related to the ATX–LPA axis in thyroid cancer

**Fig. 1:**
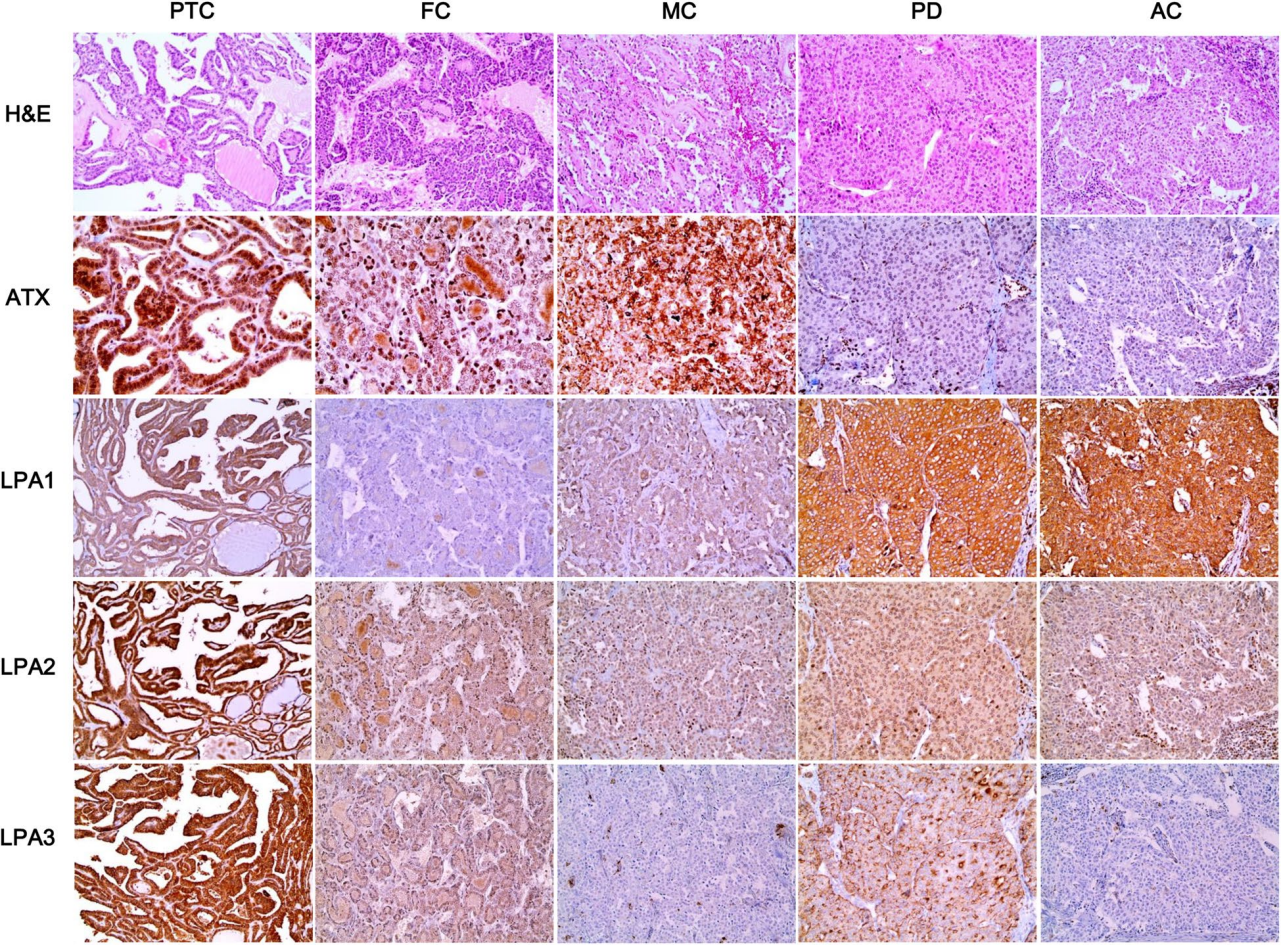
Expression of proteins related to the ATX–LPA axis in thyroid cancer. ATX is highly expressed in medullary carcinoma, as are LPA1 in PDC and AC and LPA2 and LPA3 in PTC

The expression of proteins related to the ATX–LPA axis in thyroid cancer showed differences in ATX, LPA1, LPA2, and LPA3 (p < 0.001). ATX was most highly expressed in medullary carcinoma, while LPA1 was predominant in PDC and AC, and LPA2 and LPA3 were predominant in PTC (Table 1 and Fig. 1).

**Fig. 2:**
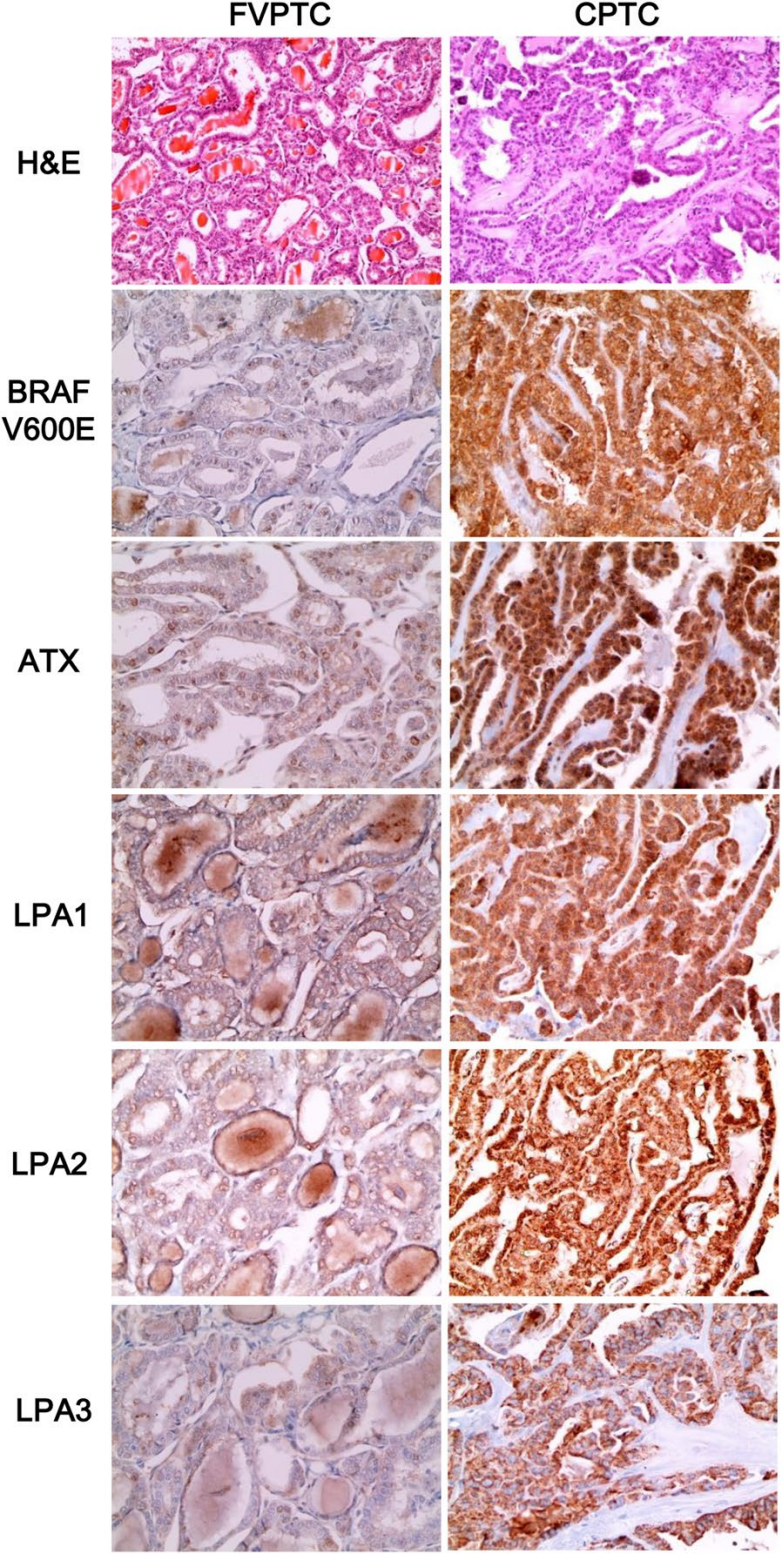
Expression of proteins related to ATX–LPA axis in TPC. ATX, LPA1, LPA2, and LPA3 expression is higher in conventional-type PTC than in FVPTC, and PTC with BRAF V600E mutation shows higher expression of those than does PTC without BRAF V600E mutation

When assessed according to the histologic subtype and BRAF V600E mutation status, ATX (p = 0.002), LPA1 (p = 0.014), and LPA2 (p = 0.001) expression was different according to the histologic subtype, and the expression of ATX, LPA1, LPA2 and LPA3 (p < 0.001) was different according to BRAF V600E mutation status. ATX, LPA1, and LPA2 expression was higher in conventional type than in FVPTC, while PTC with BRAF V600E mutation showed higher expression of ATX, LPA1, LPA2, and LPA3 than did PTC without BRAF V600E mutation (Table 2 and Fig. 2).

**Table 2:** Expression of proteins related to the ATX–LPA axis according to the histologic subtype of PTC

Parameters	Total n = 338 (%)	Histologic subtype		p-value	BRAF V600E mutation status		p-value
		Follicular variant n = 36 (%)	Conventional type n = 302 (%)		No mutation n = 102 (%)	Mutation n = 238 (%)	
ATX				*0.002*			*<* *0.001*
Negative	220 (65.1)	32 (88.9)	188 (62.3)		91 (89.2)	129 (54.7)	
Positive	118 (34.8)	4 (11.1)	114 (37.7)		11 (10.8)	107 (45.3)	
LPA1				*0.014*			*<* *0.001*
Negative	169 (50.0)	25 (69.4)	144 (47.7)		88 (86.3)	81 (34.3)	
Positive	169 (50.0)	11 (30.6)	158 (52.3)		14 (13.7)	155 (65.7)	
LPA1 (S)				0.609			0.380
Negative	323 (95.6)	35 (97.2)	288 (95.4)		99 (97.1)	224 (94.9)	
Positive	15 (4.4)	1 (2.8)	14 (4.6)		3 (2.9)	12 (5.1)	
LPA2				*0.001*			*<* *0.001*
Negative	191 (56.5)	30 (83.3)	161 (53.3)		86 (84.3)	105 (44.5)	
Positive	147 (43.5)	6 (16.7)	141 (46.7)		16 (15.7)	131 (55.5)	
LPA3				0.150			*<* *0.001*
Negative	56 (16.6)	9 (25.0)	47 (15.6)		42 (41.2)	14 (5.9)	
Positive	282 (83.4)	27 (75.0)	255 (84.4)		60 (58.8)	222 (94.1)	

Italic values represent significance of p-value (p < 0.05)

In FC cases, expression of LPA1 was highest in minimally invasive type (p = 0.004, Table 3). In PTC cases, a statistically significant positive correlation between proteins related to the ATX–LPA axis was shown in the following: LPA1 and ATX (r = 0.434, p < 0.001), LPA1 (S) and LPA1 (r = 0.187, p = 0.001), LPA2 and ATX (r = 0.459, p < 0.001), LPA2 and LPA1 (r = 0.591, p < 0.001), LPA2 and LPA1 (S) (r = 0.188, p = 0.001), LPA3 and ATX (r = 0.193, p < 0.001), LPA3 and LPA1 (r = 0.318, p < 0.001), and LPA3 and LPA2 (r = 0.295, p < 0.001) (Table 4).

**Table 3:** Expression of proteins related to the ATX–LPA axis according to histologic subtype of FC

Parameters	Total n = 111 (%)	Minimally invasive type n = 61 (%)	Encapsulated angioinvasive type n = 37 (%)	Widely invasive type n = 13 (%)	p-value
ATX					0.296
Negative	102 (91.9)	54 (88.5)	35 (94.6)	13 (100.0)	
Positive	9 (8.1)	7 (11.5)	2 (5.4)	0 (0.0)	
LPA1					*0.004*
Negative	73 (65.8)	32 (52.5)	31 (83.8)	10 (76.9)	
Positive	38 (34.2)	29 (47.5)	6 (16.2)	3 (23.1)	
LPA2					0.987
Negative	85 (76.6)	47 (77.0)	28 (75.7)	10 (76.9)	
Positive	26 (23.4)	14 (23.0)	9 (24.3)	3 (23.1)	
LPA3					0.792
Negative	52 (46.8)	27 (44.3)	19 (51.4)	6 (46.2)	
Positive	59 (53.2)	34 (55.7)	18 (48.6)	7 (53.8)	

Italic values represent significance of p-value (p < 0.05)

**Table 4:** Correlation among the expression of proteins related to the ATX–LPA axis in PTC

Parameters	ATX	LPA1	LPA1 (S)	LPA2
LPA1				
Correlation coefficient	0.434			
p-value	*<* *0.001*			
LPA1 (S)				
Correlation coefficient	0.053	0.187		
p-value	0.330	*0.001*		
LPA2				
Correlation coefficient	0.459	0.591	0.188	
p-value	*<* *0.001*	*<* *0.001*	*0.001*	
LPA3				
Correlation coefficient	0.193	0.318	0.019	0.295
p-value	*<* *0.001*	*<* *0.001*	0.731	*<* *0.001*

Italic values represent significance of p-value (p < 0.05)

### Correlations between clinicopathologic factors and expression of proteins related to the ATX–LPA axis in thyroid cancer

**Fig. 3:**
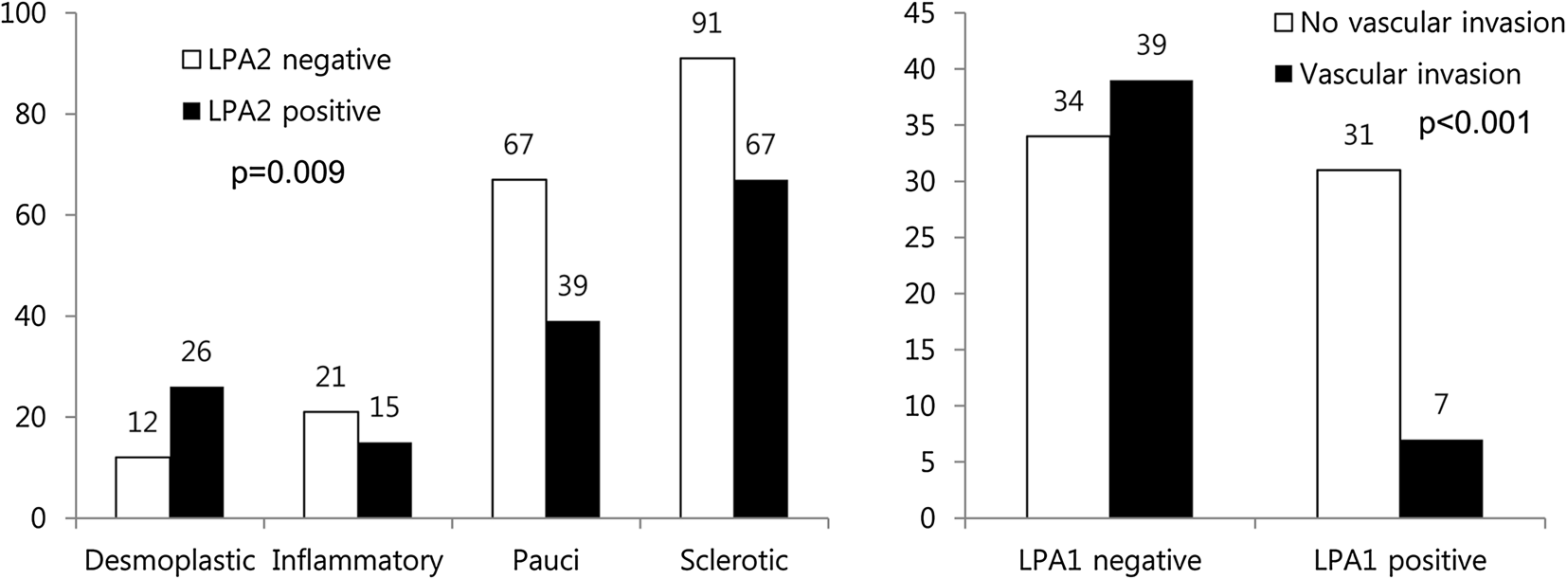
Correlations between the clinicopathologic factors and the expression of proteins related to the ATX–LPA axis in thyroid cancer. When assessed according to tumor stomal type, desmoplastic type PTC shows high expression of LPA2. In FC, vascular invasion is correlated with LPA1 negativity

When correlations between the expression of proteins related to the ATX–LPA axis and clinicopathologic factors were analyzed, LPA2 expression was different according to the stromal type (p = 0.009) in PTC, and LPA1 expression was different according to the vascular invasion status in FC (p < 0.001). In PTC, desmoplastic type showed high expression of LPA2. LPA1 negativity was correlated with vascular invasion in FC (Fig. 3).

### Impact of the expression of proteins related to the ATX–LPA axis on prognosis of thyroid cancer

**Fig. 4:**
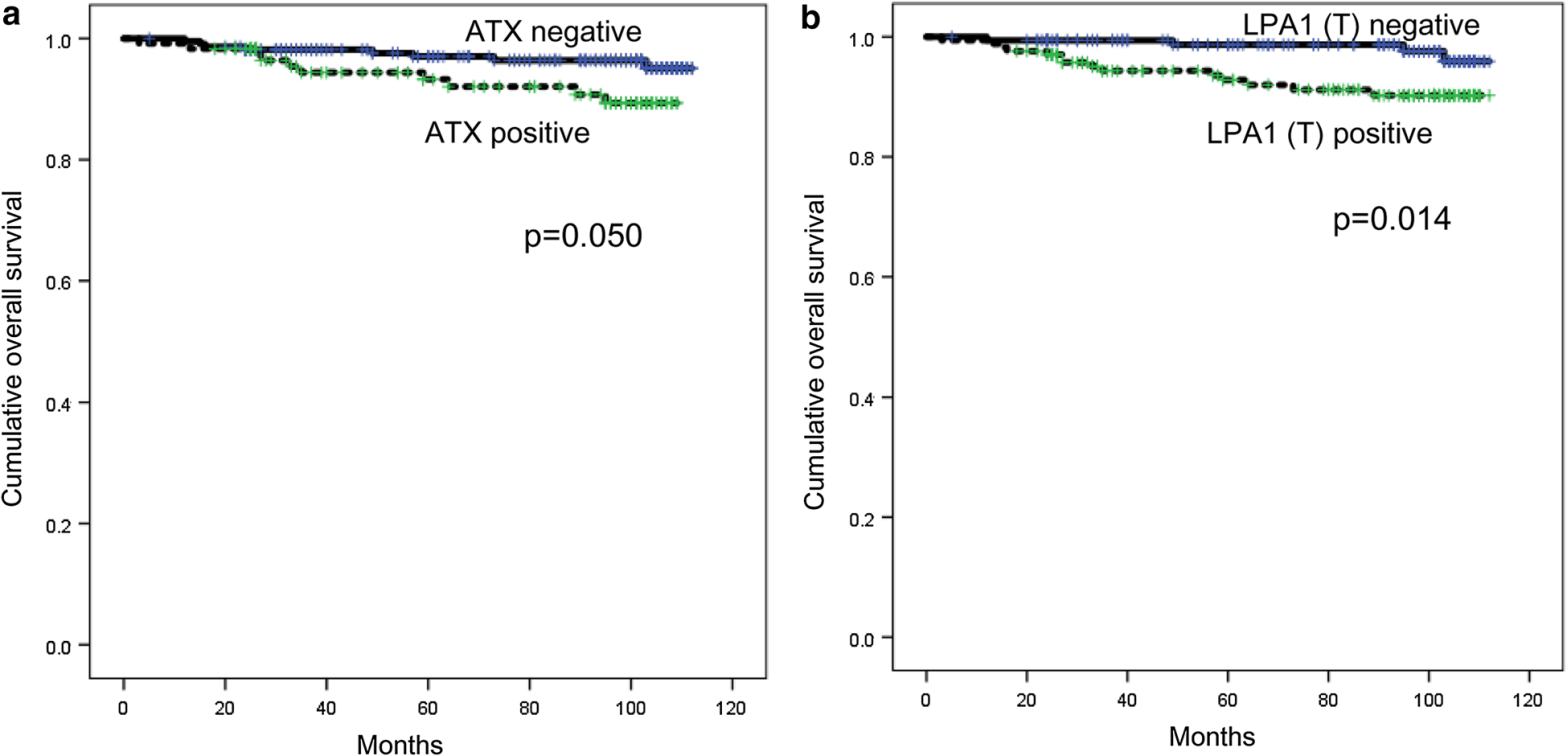
The impact of the expression of proteins related to the ATX–LPA axis on prognosis in thyroid papillary carcinoma. In univariate analysis, factors that are correlated with shorter overall survival in PTC are ATX positivity **a** and LPA1 positivity **b**

The impact of expression of proteins related to the ATX–LPA axis on the prognosis of thyroid cancer was analyzed. As a result, ATX positivity (p = 0.005) and LPA1 positivity (p = 0.014) were two factors that demonstrated correlation with shorter overall survival (Table 5 and Fig. 4) in univariate analysis. Multivariate Cox analysis showed that lymph node metastasis (hazard ratio: 5.978, 95% confidence interval (CI) 1.339–26.69; p = 0.019) was an independent factor associated with shorter DFS, while age (≥ 45 years) (hazard ratio: 16.39, 95% CI 2.129–126.2; p = 0.007) was an independent factor associated with shorter OS (Table 6).

**Table 5:** Univariate analysis of the influence of the expression of proteins related to the ATX–LPA axis in thyroid papillary cancer on disease-free and overall survival by the log-rank test

Parameter	Number of patients/recurrence/death	Disease-free survival		Overall survival	
		Mean survival (95% CI) months	p-value	Mean survival (95% CI) months	p-value
ATX			0.894		*0.050*
Negative	220/12/8	106 (103–109)		109 (107–111)	
Positive	118/6/10	104 (101–108)		102 (98–106)	
LPA1			0.670		*0.014*
Negative	169/10/4	106 (103–109)		110 (109–112)	
Positive	169/8/14	107 (104–110)		105 (101–108)	
LPA1 (S)			0.156		0.909
Negative	323/16/17	107 (105–109)		108 (106–109)	
Positive	15/2/1	96 (79–113)		102 (89–116)	
LPA2			0.947		0.316
Negative	191/10/8	107 (104–110)		108 (106–111)	
Positive	147/8/10	106 (103–110)		106 (103–110)	
LPA3			0.209		0.204
Negative	56/1/1	108 (104–111)		108 (106–111)	
Positive	282/17/17	106 (103–109)		107 (105–109)	

Italic values represent significance of p-value (p < 0.05)

**Table 6:** Multivariate analysis of factors influencing survival of patients with PTC

Included parameters	Disease-free survival			Overall survival		
	Hazard ratio	95% CI	p-value	Hazard ratio	95% CI	p-value
Age (years)			0.617			*0.007*
< 45 versus ≥ 45	1.282	0.485–3.389		16.39	2.129–126.2	
Sex			0.508			0.925
Male versus female	0.700	0.244–2.011		1.065	0.290–3.915	
Tumor size (cm)			0.816			0.188
≤ 2.0 versus > 2.0	1.134	1.134–3.268		2.005	0.711–5.653	
Tumor extension			0.378			0.440
Intrathyroidal versus extrathyroidal	0.641	0.239–1.722		0.647	0.214–1.955	
LN metastasis			*0.019*			0.064
No versus yes	5.978	1.339–26.69		2.940	0.939–9.202	
ATX			0.997			0.360
Negative versus positive	0.998	0.334–2.981		1.633	0.571–4.665	
LPA1			0.688			0.252
Negative versus positive	0.805	0.278–2.327		2.093	0.592–7.403	

Italic values represent significance of p-value (p < 0.05)

## Discussion

We investigated the expression of proteins related to the ATX–LPA axis in thyroid cancer. First, ATX was more highly expressed in medullary carcinoma than in other subtypes. Previous studies have shown that ATX-related protein expression was higher in thyroid cancer or metastatic thyroid cancer than in normal thyroid tissue or benign thyroid neoplasm [9, 10], but there has not been any report published to date on ATX-related protein expression in thyroid medullary carcinoma. The possible mechanism for the high expression of ATX in thyroid medullary carcinoma is the JAK/STAT3 pathway. In previous studies on breast cancer, activation of the JAK/STAT3 pathway was reported to increase the expression of ATX [12], which is suggested to represent a putative STAT3 target gene. Meanwhile, it has been reported that the JAK/STAT3 pathway is also activated in thyroid medullary carcinoma [13, 14]; consequently, it is only plausible that ATX expression is somehow associated with the JAK/STAT3 pathway. Our study showed that LPA1 was highly expressed in PDC and AC, and that LPA2 and LPA3 were similarly highly expressed in PTC. A previous study on thyroid cancer reported that LPA1 messenger RNA expression showed no difference among normal thyroid tissue, benign thyroid nodule, and thyroid cancer [15]. However, the study had included PTC and FC for thyroid cancer; therefore, its direct comparison with our study, which includes PDC and AC in addition to PTC and FC for thyroid cancer, is not possible. Separately, another previous study suggested CD97 as a dedifferentiation marker in thyroid cancer [16], the expression of which is reported to be correlated with LPA receptor in thyroid cancer [17]. Such a result is related with the high expression of LPA1 in PDC and AC in our study. LPA2 has been previously reported to be highly expressed in PTC, which is in concordance with our results [15]. On the other hand, our study showed low expression of proteins related to the ATX–LPA axis in PTC and FVPTC without BRAF V600E mutation, a result that disagreed with those of the previous study that reported that ATX protein expression is not correlated with BRAF mutation status [9]. Usually, most FVPTC cases do not harbor BRAF V600E mutation; thus, it is necessary to assess the correlation between BRAF V600E mutation and the ATX–LPA axis. The most plausible mechanism is that BRAF V600E mutation activates cytokine release, which in turn increases ATX protein expression. Tumor cells showing BRAF V600E mutation tend to have increased cytokine secretion such as that of interleukin (IL)-1β, IL-6, and IL-8 [18], because cytokines such as tumor necrosis factor alpha or IL-1β increase ATX and LPA levels. In our study, ATX and LPA1 expression was a poor prognosis factor in PTC, which is in concordance with previous study results that had identified ATX as a poor prognosis factor in breast cancer [19] and in prostate cancer [20] and, separately, LPA2 as a poor prognosis factor in breast cancer [21].

The clinical implication of our study is that the ATX–LPA signaling axis may be a possible therapeutic target in thyroid cancer. Ki16425, a nonlipid competitive inhibitor of LPA1 and LPA3, actually suppressed bone metastasis in a mouse model [22]. Debio0719, an R-stereoisomer of Ki16425, was found to inhibit distant metastasis [23, 24]. BrP-LPA, which is a dual ATX and pan-LPAR inhibitor, inhibited cell migration and invasion [25]. In addition, an ATX inhibitor, ONO-843050, decreased tumor volume to 50% to 60% in a mouse model of PTC [9]. Therefore, the ATX–LPA axis may be an effective treatment target for thyroid cancer, and further clinical trials are necessary.

## Conclusion

The expression of proteins related to the ATX–LPA axis is different according to tumor subtype and is notably higher in metastatic thyroid cancer than in primary tumor. Furthermore, proteins related to the ATX–LPA axis may represent candidates for effective treatment target in thyroid cancer.

## Supplementary information

**Additional file 1.** Additional tables.

## Data Availability

All data generated and analyzed during this study are included in this published article.

## Abbreviations

ATX: autotaxin
LPC: lysophosphatidylcholine
LPA: lysophosphatidate
PTC: papillary thyroid carcinoma
FC: follicular carcinoma
MC: medullary carcinoma
PDC: poorly differentiated carcinoma
AC: anaplastic carcinoma
