# Supplementary material for: Expression of proteins related to autotaxin–lysophosphatidate signaling in thyroid tumors
Source: J Transl Med. 2019 Aug 28;17:288. doi: 10.1186/s12967-019-2028-7 (PMC6712878; doi:10.1186/s12967-019-2028-7)
Supplement: Supplementary file 1 — Additional file 1. Additional tables. [file 12967_2019_2028_MOESM1_ESM.docx]

Table S1. Source, clone, and dilution of antibodies used in this study

| **Antibody** | **Company** | | | **Clone** | **Dilution** | |  |
| --- | --- | --- | --- | --- | --- | --- | --- |
| ATX | | Abcam, Cambridge, UK | Polyclonal | | | 1:1,000 | |
| LPA1 | | Abcam, Cambridge, UK | EPR9710 | | | 1:100 | |
| LPA2 | | Abcam, Cambridge, UK | Polyclonal | | | 1:100 | |
| LPA3 | | Abcam, Cambridge, UK | Polyclonal | | | 1:250 | |
| BRAF V600E | Ventana, Tucson, AZ, USA | | | VE1 | 1:50 | |  |

| Table S2. Basal characteristics of patients with papillary thyroid carcinoma | | | | | | | |
| --- | --- | --- | --- | --- | --- | --- | --- |
| Parameters | Total  n = 338 (%) | Histologic subtype | | p-value | BRAF V600E mutation status | | p-value |
|  |  | Follicular variant  n = 36 (%) | Conventional type  n = 302 (%) |  | No mutation  N = 102 (%) | Mutation  N = 236 (%) |  |
| Age (years) |  |  |  | 0.774 |  |  | 0.089 |
| < 45 | 152 (45.0) | 17 (47.2) | 135 (44.7) |  | 53 (52.0) | 99 (41.9) |  |
| ≥ 45 | 186 (55.0) | 19 (52.8) | 167 (55.3) |  | 49 (48.0) | 137 (58.1) |  |
| Sex |  |  |  | 0.702 |  |  | 0.155 |
| Male | 67 (19.8) | 8 (22.2) | 59 (19.5) |  | 25 (24.5) | 42 (17.8) |  |
| Female | 271 (80.2) | 28 (77.8) | 243 (80.5) |  | 77 (75.5) | 194 (82.2) |  |
| Tumor size (cm) |  |  |  | 0.950 |  |  | 0.428 |
| ≤ 2.0 | 266 (78.7) | 28 (77.8) | 238 (78.8) |  | 76 (74.5) | 190 (80.5) |  |
| > 2.0 and ≤ 4.0 | 65 (19.2) | 7 (19.4) | 58 (19.2) |  | 23 (22.5) | 42 (17.8) |  |
| > 4.0 | 7 (2.1) | 1 (2.8) | 6 (2.0) |  | 3 (2.9) | 4 (1.7) |  |
| Tumor margin |  |  |  | **0.009** |  |  | **0.009** |
| Infiltrative | 285 (84.3) | 25 (69.4) | 260 (86.1) |  | 78 (76.5) | 207 (87.7) |  |
| Expanding | 53 (15.7) | 11 (30.6) | 42 (13.9) |  | 24 (23.5) | 29 (12.3) |  |
| Tumor extension |  |  |  | 0.958 |  |  | 0.406 |
| Intrathyroidal | 102 (30.2) | 11 (30.6) | 91 (30.1) |  | 34 (33.3) | 68 (28.8) |  |
| Extrathyroidal | 236 (69.8) | 25 (69.4) | 211 (69.9) |  | 68 (66.7) | 168 (71.2) |  |
| Histologic subtype |  |  |  |  |  |  | **< 0.001** |
| Conventional |  |  |  |  | 21 (20.6) | 15 (6.4) |  |
| Follicular |  |  |  |  | 81 (79.4) | 221 (93.6) |  |
| LN metastasis |  |  |  | 0.533 |  |  | 0.178 |
| No | 134 (36.9) | 16 (44.4) | 118 (39.1) |  | 46 (45.1) | 88 (37.7) |  |
| Yes | 204 (60.4) | 20 (55.6) | 184 (60.9) |  | 56 (54.9) | 148 (62.7) |  |
| Distant metastasis |  |  |  | 0.948 |  |  | 0.408 |
| No | 320 (94.7) | 34 (94.4) | 286 (94.7) |  | 95 (93.1) | 225 (95.3) |  |
| Yes | 18 (5.3) | 2 (5.6) | 16 (5.3) |  | 7 (6.9) | 11 (4.7) |  |

| Table S3. Basal characteristics of patients with follicular carcinoma (FC) | | | | | | |
| --- | --- | --- | --- | --- | --- | --- |
| Parameters | Total  n = 111 (%) | Minimally invasive type  n = 61 (%) | Encapsulated angioinvasive type n = 37 (%) | Widely invasive type  n =13 (%) | p-value |  |
| Age (years) |  |  |  |  | 0.334 |  |
| < 45 | 50 (45.0) | 31 (50.8) | 15 (40.5) | 4 (30.8) |  |  |
| ≥ 45 | 61 (55.0) | 30 (49.2) | 22 (59.5) | 9 (69.2) |  |  |
| Sex |  |  |  |  | 0.272 |  |
| Male | 28 (25.2) | 12 (19.7) | 11 (29.7) | 5 (38.5) |  |  |
| Female | 83 (74.8) | 49 (80.3) | 26 (70.3) | 8 (61.5) |  |  |
| Tumor size (cm) |  |  |  |  | 0.040 |  |
| ≤ 2.0 | 34 (30.6) | 23 (37.7) | 11 (29.7) | 0 (0.0) |  |  |
| > 2.0 and ≤ 4.0 | 48 (43.2) | 27 (44.3) | 13 (35.1) | 8 (61.5) |  |  |
| > 4.0 | 29 (26.1) | 11 (18.0) | 13 (35.1) | 5 (38.5) |  |  |
| Capsular invasion |  |  |  |  | < 0.001 |  |
| No | 13 (11.7) | 0 (0.0) | 13 (35.1) | 0 (0.0) |  |  |
| Yes | 98 (88.3) | 61 (100.0) | 24 (64.9) | 13 (100.0) |  |  |
| Vascular invasion |  |  |  |  | < 0.001 |  |
| No | 65 (58.6) | 61 (100.0) | 0 (0.0) | 4 (30.8) |  |  |
| Yes | 46 (41.4) | 0 (0.0) | 37 (100.0) | 9 (69.2) |  |  |
| Tumor extension |  |  |  |  | < 0.001 |  |
| Intrathyroidal | 94 (84.7) | 53 (86.9) | 35 (94.6) | 6 (46.2) |  |  |
| Extrathyroidal | 17 (15.3) | 8 (13.12) | 2 (5.4) | 7 (53.8) |  |  |
| LN metastasis |  |  |  |  | 0.147 |  |
| No | 109 (98.2) | 61 (100.0) | 36 (97.3) | 12 (92.3) |  |  |
| Yes | 2 (1.8) | 0 (0.0) | 1 (2.7) | 1 (7.7) |  |  |
| Distant metastasis |  |  |  |  | 0.001 |  |
| No | 100 (90.1) | 58 (95.1) | 34 (91.9) | 8 (61.5) |  |  |
| Yes | 11 | 3 (4.9) | 3 (8.1) | 5 (38.5) |  |  |

| Table S4. Basal characteristics of patients with MC, PDC, or AC | | | |
| --- | --- | --- | --- |
| Parameters | MC, n = 69 (%) | PDC, n = 23 (%) | AC, n = 4 (%) |
| Age (years) |  |  |  |
| < 45 | 21 (30.4) | 4 (17.4) | 0 (0.0) |
| ≥ 45 | 48 (69.6) | 19 (82.6) | 4 (100.0) |
| Sex |  |  |  |
| Male | 22 (31.9) | 10 (43.5) | 0 (0.0) |
| Female | 47 (68.1) | 13 (56.5) | 4 (100.0) |
| Tumor size (cm) |  |  |  |
| ≤ 2.0 | 52 (75.4) | 8 (34.8) | 0 (0.0) |
| > 2.0 and ≤ 4.0 | 14 (20.3) | 9 (39.1) | 0 (0.0) |
| > 4.0 | 3 (4.3) | 6 (26.1) | 4 (100.0) |
| Tumor margin |  |  |  |
| Infiltrative | 44 (63.8) | 17 (73.9) | 4 (100.0) |
| Expanding | 25 (36.2) | 6 (26.1) | 0 (0.0) |
| Tumor extension |  |  |  |
| Intrathyroidal | 51 (73.9) | 11 (47.8) | 0 (0.0) |
| Extrathyroidal | 18 (26.1) | 12 (52.2) | 4 (100.0) |
| LN metastasis |  |  |  |
| No | 46 (66.7) | 22 (95.7) | 2 (50.0) |
| Yes | 23 (33.3) | 1 (4.3) | 2 (50.0) |
| Distant metastasis |  |  |  |
| No | 66 (95.7) | 16 (69.6) | 0 (0.0) |
| Yes | 3 (4.3) | 7 (30.4) | 4 (100.0) |
